# Further Evidence of Increasing Diversity of *Plasmodium vivax* in the Republic of Korea in Recent Years

**DOI:** 10.1371/journal.pone.0151514

**Published:** 2016-03-18

**Authors:** Jung-Yeon Kim, Youn-Kyoung Goo, Young-Gun Zo, So-Young Ji, Hidayat Trimarsanto, Sheren To, Taane G. Clark, Ric N. Price, Sarah Auburn

**Affiliations:** 1 Division of Malaria and Parasitic Diseases, National Institute of Health, Korea CDC, Osong Saeng-myeong, 2 ro, Osong Health Technology Administration, Osong, Republic of Korea; 2 Department of Parasitology and Tropical Medicine, Kyungpook National University School of Medicine, Daegu, 700–422, Republic of Korea; 3 Department of Molecular Parasitology, Sungkyunkwan University School of Medicine and Center for Molecular Medicine, Samsung Biomedical Research Institute, Suwon, Gyeonggi-do 440–746, Republic of Korea; 4 Eijkman Institute for Molecular Biology, Jl. Diponegoro 69, Jakarta Pusat, 10430, Indonesia; 5 Agency for Assessment and Application of Technology, Jl. MH Thamrin 8, Jakarta, 10340, Indonesia; 6 Global and Tropical Health Division, Menzies School of Health Research and Charles Darwin University, Darwin, NT 0810, Australia; 7 Faculty of Infectious and Tropical Diseases, London School of Hygiene and Tropical Medicine, Keppel Street, London, WC1E 7HT, United Kingdom; 8 Faculty of Epidemiology and Population Health, London School of Hygiene and Tropical Medicine, Keppel Street, London, WC1E 7HT, United Kingdom; 9 Centre for Tropical Medicine and Global Health, Nuffield Department of Clinical Medicine, University of Oxford, Oxford, United Kingdom; Ehime University, JAPAN

## Abstract

**Background:**

Vivax malaria was successfully eliminated from the Republic of Korea (ROK) in the late 1970s but re-emerged in 1993. Two decades later as the ROK enters the final stages of malaria elimination, dedicated surveillance of the local *P*. *vivax* population is critical. We apply a population genetic approach to gauge *P*. *vivax* transmission dynamics in the ROK between 2010 and 2012.

**Methodology/Principal Findings:**

*P*. *vivax* positive blood samples from 98 autochthonous cases were collected from patients attending health centers in the ROK in 2010 (*n* = 27), 2011 (*n* = 48) and 2012 (*n* = 23). Parasite genotyping was undertaken at 9 tandem repeat markers. Although not reaching significance, a trend of increasing population diversity was observed from 2010 (*H*_E_ = 0.50 ± 0.11) to 2011 (*H*_E_ = 0.56 ± 0.08) and 2012 (*H*_E_ = 0.60 ± 0.06). Conversely, linkage disequilibrium declined during the same period: *I*_AS_ = 0.15 in 2010 (*P* = 0.010), 0.09 in 2011 (*P* = 0.010) and 0.05 in 2012 (*P* = 0.010). In combination with data from other ROK studies undertaken between 1994 and 2007, our results are consistent with increasing parasite divergence since re-emergence. Polyclonal infections were rare (3% infections) suggesting that local out-crossing alone was unlikely to explain the increased divergence. Cases introduced from an external reservoir may therefore have contributed to the increased diversity. Aside from one isolate, all infections carried a short MS20 allele (142 or 149 bp), not observed in other studies in tropical endemic countries despite high diversity, inferring that these regions are unlikely reservoirs.

**Conclusions:**

Whilst a number of factors may explain the observed population genetic trends, the available evidence suggests that an external geographic reservoir with moderate diversity sustains the majority of *P*. *vivax* infection in the ROK, with important implications for malaria elimination.

## Introduction

Across the globe, *P*. *vivax* infection is recognised as a major cause of morbidity and in some locations associated with mortality [1]. The direct and indirect morbidity and associated mortality of *P*. *vivax* has a massive impact on the poorest communities of malarious countries. Conservative estimates of the overall global cost of *P*. *vivax* infection to the individual in terms of lost productivity, healthcare costs and transport to clinics are between $1.4 and 4.0 billion per year[2]. The clinical and economic burden and the rise of *P*. *vivax* resistant to chloroquine, the first-line treatment against the blood stages of vivax malaria in many endemic countries, emphasize the importance of containing and eliminating the parasite [3].

Since the re-emergence of malaria in 1993 [4], the Republic of Korea (ROK) has faced significant challenges in its endeavour to contain the spread of *P*. *vivax* infection and ultimately achieve malaria-free status again. In the early phase of the re-emergence, malaria cases were observed primarily in military personnel and veterans deployed near the demilitarized zone bordering the Democratic People’s Republic of Korea (DPRK). However in the subsequent two decades the proportion of *P*. *vivax* infections in the civilian population has gradually increased, as has the incidence of cases in southern parts of the country [5]. With increasing population movements for social and economic purposes, the risks of drug resistance importation from overseas as well as along border regions, present an important threat to public health. A recent study in the ROK highlighted evidence of local *P*. *vivax* infection resistant to chloroquine [6], possibly reflecting importation from areas of established parasite drug resistance.

As the ROK enters the malaria pre-elimination phase, dedicated surveillance of the parasite population is critical [7]. During this phase, strategies are needed to identify the remaining pockets of infection and their major reservoirs, to detect and characterize imported cases, monitor the efficacy of ongoing interventions, and identify emerging outbreaks as early as possible. Previous studies in the ROK conducted between 1994 and 2007, have demonstrated the utility of polymorphic short-tandem repeat (STR) markers such as microsatellites to inform on the transmission dynamics of the local parasite population [8–10]. Indeed, STR-based approaches have been increasingly applied to population genetic studies of *P*. *vivax*, with useful insights derived into local transmission dynamics in a broad range of endemic settings [11–30].

Using a panel of nine standard STR markers selected for use by the Asia Pacific Malaria Elimination Network (APMEN) Vivax Working Group [31], we genotyped a selection of autochthonous and imported *P*. *vivax* infections collected in the ROK between 2010 and 2012. We describe the local population diversity and structure, including comparison to prior molecular analyses in the ROK.

## Methods

### Ethics

All samples were collected with written informed consent from the patient, parent or legal guardian (for individuals less than 18 years of age). The study was approved by the Korea Centers for Disease Control and Prevention Institutional Review Board, Republic of Korea (Protocol No. 2011-02CON-14-P) and the Human Research Ethics Committee of Northern Territory Department of Health and Menzies School of Health Research (HREC-2012-1895).

### Study Sites and Sample Collection

**Fig 1:**
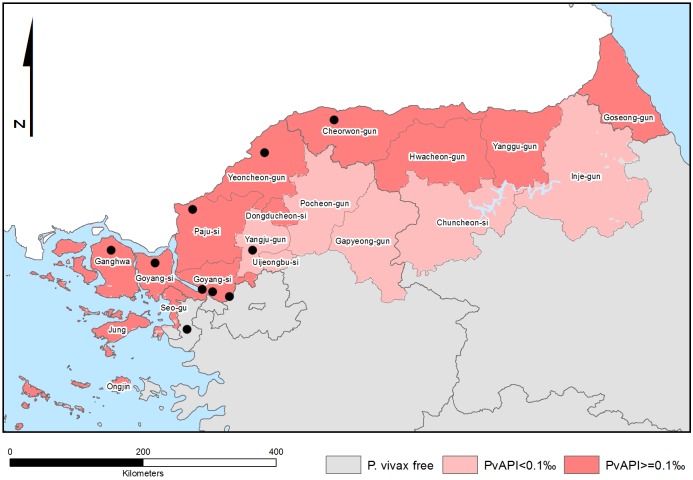
Locations at which infections were acquired. This map was generated by the Malaria Atlas Project, University of Oxford. The colour scale reflects the model-based geostatistical point estimates of the annual mean *P*. *vivax* parasite rate in the 1–99 year age range (*Pv*PR) within the stable spatial limits of *P*. *vivax* transmission in 2010 [32]. The approximate locations of the study sites are indicated with black dots. All MAP maps are available to users under the CCAL 3.0. http://www.map.ox.ac.uk/about-map/open-access/.

Approximately 5 ml venous blood samples preserved in EDTA-coated vacutainers were collected from 101 patients with *P*. *vivax* positive infection confirmed by microscopy of Giemsa-stained blood films, presenting to local health centers and hospitals across the ROK between April 2010 and September 2012. Fig 1 illustrates the administrative regions from which samples were sourced. All blood samples were stored in a fridge or freezer and transported to the Korea Centres for Disease Control (KCDC) in Seoul within 24 hours of collection. Details on recent travel history and home address were recorded for each patient in order to determine the most likely location at which the infection was acquired.

### Molecular Processing

Genomic DNA extraction was undertaken at the KCDC. Extractions were performed on 200 μl of whole blood using the QIAamp DNA Mini kit (Qiagen, USA) according to the manufacturer’s protocol. Nested PCR was performed to confirm the microscopy diagnosis of vivax malaria [33].

*P*. *vivax* genotyping was undertaken at 9 previously described short tandem repeat markers selected as a consensus panel for genotyping studies in the APMEN Vivax Working Group. The marker selection comprises Pv3.27, msp1F3, MS1, MS5, MS8, MS10, MS12, MS16 and MS20 [19, 34]. In addition to analyses on the full spectrum of 9 markers, investigations were undertaken on a subset of 5 markers (MS1, MS5, MS10, MS12 and MS20) defined as exhibiting balanced diversity in a recent investigation of the correlation between diversity and local endemicity in a panel of commonly used *P*. *vivax* STR markers [35]. This study demonstrated that the markers with balanced diversity were optimal for characterising population structure, whilst markers with excess diversity such as MS16, pv3.27 and MS8 had enhanced ability to identify polyclonal infections. The Pv3.27, MS16 and msp1F3 loci were amplified using the methods described by Abdullah *et al*. [11]. The MS1, MS5, MS8, MS10, MS12 and MS20 loci were amplified using a single round of PCR following the protocol described by Gunawardena *et al*. [16]. The primer sequences and chromosomal locations used for each marker are detailed in Abdullah *et al*. [11].

The final labelled PCR products were diluted and sized by denaturing capillary electrophoresis at Charles Darwin University on an ABI 3100 Genetic Analyzer with GeneScan LIZ-600 (Applied Biosystems) internal size standards. Genotype calling was facilitated with GeneMapper Version 4.0. In order to reduce potential artefacts from background noise or stutter, an arbitrary fluorescent intensity threshold of 100 rfu was applied for peak detection. All electropherogram traces were also inspected manually. For each isolate, at each locus, the predominant allele (highest intensity peak), and any additional alleles with peak height at least one-third of the height of the predominant allele were scored [36]. Genotyping success was defined as the presence of at least one allele at a given locus in a given sample.

### Population Genetic Analysis

Since asexual *P*. *vivax* stages are haploid, an infection was defined as polyclonal if more than one allele was observed at one or more loci. The Multiplicity of Infection (MOI) for a given sample was defined as the maximum number of alleles observed at any of the loci investigated. The mean MOI was calculated from the individual sample MOIs for each study site. With the exception of the MOI calculations, in all analyses, only the predominant allele at each locus in each isolate was used [36].

The expected heterozygosity (*H*_E_) was measured as an index of population diversity. *H*_E_ was calculated for each locus using the formula *H*_E_ = [*n*/ (*n*-1)] [1-Σ*p*_*i*_^2^], where *n* is the number of isolates analyzed and *pi* is the frequency of the *ith* allele in the population. The correction factor *n*/(*n*-1) was included to enhance comparison between populations with differing sample size.

The pairwise *F*_ST_ metric was used to gauge the genetic distance between populations. Calculations were undertaken using Arlequin software (version 3.5) [37]. In addition to the classic *F*_ST_ metric, standardized measures of the genetic distance (*F’*_ST_) were calculated to adjust for high marker diversity and enable greater comparability with other studies [38]. The *F’*_ST_ provides a measure of *F*_ST_ expressed as a fraction of the maximum possible value of this statistic, whereby *F’*_ST_ = *F*_ST_ /*F*_ST_−max. *F*_ST_−max was calculated by recoding the data to obtain the maximum divergence among populations.

Population structure was further assessed using STRUCTURE software version 2.3.3 to determine the most likely number of populations (*K*) and ancestry of each isolate to the *K* populations [39]. Twenty replicates, with 10,000 iterations (10,000 burn-in) and were run for each of *K* from 1–10 using the model parameters of admixture with correlated allele frequencies. The most probable *K* was derived by calculating *ΔK* as described elsewhere [40] for each of *K* = 2–8. Barplots illustrating the ancestry of each isolate to each of the *K* populations were prepared using *distruct* software version 1.1 [41].

Multi-locus genotypes (or infection haplotypes) were reconstructed from the predominant allele at each locus in isolates with no missing data at any of the loci investigated. Using these multi-locus genotypes, linkage disequilibrium (LD) was measured by the standardised index of association (*I*_A_^S^) using the web-based LIAN 3.5 software [42]. Under the null hypothesis of linkage equilibrium, the significance of the *I*_A_^S^ estimates was assessed using 10,000 random permutations of the data. LD was assessed in 1) the full sample set and 2), for assessment of epidemic transmission, with each unique haplotype represented just once.

Using the multi-locus genotypes described above, the genetic relatedness between sample pairs was assessed by measuring the proportion of alleles shared between haplotype pairs (*ps*). Using (1-*ps*) as a measure of genetic distance [43], an unrooted neighbour-joining tree [44] was generated with the APE (Analysis of Phylogenetics and Evolution) package in R [45]. Suspected imported cases were included in the neighbour-joining analysis only.

### Statistical Tests

Comparison of the patient age distributions between study years were undertaken using the Wilcoxon rank sum test with continuity correction. The Kruskal-Wallis test was used to compare the expected heterozygosity (*H*_E_) between study years. Assessment of the significance of the difference in the proportion of male patients between the study years was determined using Pearson's Chi-squared test with Yates' continuity correction. All statistical tests were performed using R software [46]. Significance was determined at an alpha of 0.05.

## Results

### Samples and Genotyping

Between January 2010 and December 2012, a total of 101 parasite isolates were collected from patients infected with *P*. *vivax*, three of whom had a travel history consistent with imported infections from Cambodia, Brazil and Ethiopia. Patient and parasitological details for the parasite isolates are summarised in Table 1. Amongst the 98 patients with autochthonous infections, 64 (65%) were male, with no significant difference observed between study years (*P* <0.05). All three patients with likely imported cases were male. The median (range) age of patients with autochthonous infections was 45 years (12–74 years), with no significant differences observed between years (*P* <0.05).

**Table 1:** Sample details.

Year	No. patients	No. males (%)	Median patient age, years (range) *
2010	27	18 (67%)	49 (12–63)
2011	48	33 (69%)	43 (15–74)
2012	23	13 (57%)	47 (20–73)
2010–12	98	64 (65%)	45 (12–74)

* Details on median patient age were missing for 9, 20 and 11 individuals in 2010, 2011 and 2012 respectively.

Details of the parasite genotypes at each locus are presented in the supplementary material (S1 Table). After the exclusion of 2 autochthonous ROK isolates with high genotype failure (7 and 9 failed markers), 96 (98%) ROK samples and 3 (100%) imported cases (with at most 2 genotype fails) were available for molecular analysis. Amongst these, all 3 imported samples (100%) and 77 (80%) ROK isolates, including 24 (89%) from 2010, 34 (74%) from 2011 and 19 (83%) from 2012, exhibited successful calls at all nine markers, enabling the reconstruction of multi-locus genotypes (MLGs) for neighbour-joining and LD analyses. All markers displayed less than 10% genotyping failures (range: 0–8% fails) (S2 Table).

### Polyclonality and Population Diversity

A total of 3.1% (3/96) ROK samples (*n* = 1 in 2010, *n* = 1 in 2011 and *n* = 1 in 2012) were polyclonal. The complexity of the polyclonal infections was low, in each case with only one locus displaying evidence of multiple alleles, and a maximum of 2 alleles observed at any given locus. This was reflected in an overall mean MOI of 1.03 clones per infection (1.04 in 2010, 1.02 in 2011 and 1.04 in 2012). On adjusting the relative threshold for calling minor alleles from 33% to 10%, 6.2% (6/96) ROK samples were polyclonal.

At the population level, the average diversity across 2010 to 2012 was moderate (*H*_E_ = 0.56; Table 2). When stratified by year, a moderate increase was observed in the expected heterozygosity from *H*_E_ = 0.5 in 2010 to 0.56 in 2011 and 0.60 in 2012, although this did not reach statistical significance (*P*<0.05). In comparison with other microsatellite-based studies where the diversity of *P*. *vivax* in the ROK was assessed in earlier years [8, 10], our results demonstrate a continual increase in diversity from 1994 to 2013 (Table 3).

**Table 2:** Marker diversity by year between 2010 and 2013.

	2010–12	2010	2011	2012	
Marker	*H*_E_	*H*_E_	*H*_E_	*H*_E_	Pattern of change
**MS5**	0.79	0.80	0.79	0.80	Stable
**MS16**	0.79	0.75	0.80	0.75	Fluctuation
**pv3.27**	0.75	0.78	0.76	0.72	Decline
**MS1**	0.73	0.77	0.71	0.76	Fluctuation
**msp1f3**	0.73	0.70	0.76	0.74	Fluctuation
**MS8**	0.36	0.21	0.37	0.44	Increase
**MS10**	0.35	0.26	0.38	0.44	Increase
**MS12**	0.31	0.26	0.33	0.36	Increase
**MS20**	0.19	0	0.14	0.42	Increase
^a^ **9 MS Mean ± se**	0.56 ± 0.08	0.50 ± 0.11	0.56 ± 0.08	0.60 ± 0.06	-
^b^ **5 MS Mean ± se**	0.48 ± 0.12	0.42 ± 0.16	0.47 ± 0.12	0.56 ± 0.09	-

*H*_E_ = expected heterozygosity.

^a^ All 9 markers used in analysis.

^b^ Analysis restricted to 5 markers defined as balanced by Sutton [35]: MS1, MS5, MS10, MS12, MS20.

**Table 3:** Population diversity and LD in the ROK between 1994 and 2013.

Study	No. samples	No. markers	Collection years	*H*_E_ ± SE	Multilocus LD, *I*_SA_ (*P*-value)
Iwagami et al. [10]	81	14 ^a^	1994–1998	0.32 ± 0.04	0.584 (*P* < 0.001)
Honma et al. [8]	29	13 ^b^	1997–2000	0.38 ± 0.06	0.557 (*P* < 0.001)
Iwagami et al. [10]	53	14 ^a^	1999–2003	0.41 ± 0.03	0.315 (*P* < 0.001)
Iwagami et al. [10]	29	14 ^a^	2004–2008	0.50 ± 0.05	0.140 (*P* < 0.001)
Honma et al. [8]	29	13 ^b^	2007	0.55 ± 0.06	0.181 (*P* < 0.001)
This study	96	9 ^c^	2010–12	0.56 ± 0.08	0.098 (*P* < 0.01)

^a^ MS1, MS2, MS3, MS4, MS5, MS6, MS7, MS8, MS9, MS10, MS12, MS15, MS16, MS20.

^b^ pv1.501, pv3.27, pv3.502, pv14.297, MS3, MS5, MS6, MS8, MS9, MS10, MS16, MS15, MS16, MS20.

^c^ pv3.27, msp1F3, MS1, MS5, MS8, MS10, MS12, MS16, MS20.

Whilst differences were observed in the dynamics of individual markers over time in our study, the four markers with the overall lowest diversity (MS8, MS10, MS12 and MS20; all *H*_E_ <0.5) all demonstrated moderate increases in diversity from 2010 to 2011 and 2012. These 4 markers also happened to be amongst the set of 5 markers defined as having balanced diversity [35]. Although the overall diversity was lower using the 5 balanced markers (*H*_E_ = 0.48) versus the 9 markers (*H*_E_ = 0.56), the trend of increasing diversity from 2010 (*H*_E_ = 0.42) to 2011 (*H*_E_ = 0.47) and 2012 (*H*_E_ = 0.56) was maintained (*P*<0.05) (Table 2).

Relative to other geographic regions investigated in our other APMEN studies, the MS20 locus was notable not just in its low diversity in the ROK but also its moderately distinct allele size range in this population (S4 Table). Other than a single ROK infection displaying MS20 allele 175, all isolates carried relatively short alleles, namely alleles 142 or 149. Neither of these short MS20 alleles were observed in any of the imported cases investigated in this study (alleles 194, 218 and 233 observed) or in our other studies conducted in Malaysia [11], Indonesia [25], Ethiopia [13] or Bhutan (manuscript under review), despite moderate to high diversity at the locus in all of these sites.

### Parasite Relatedness

**Fig 2:**
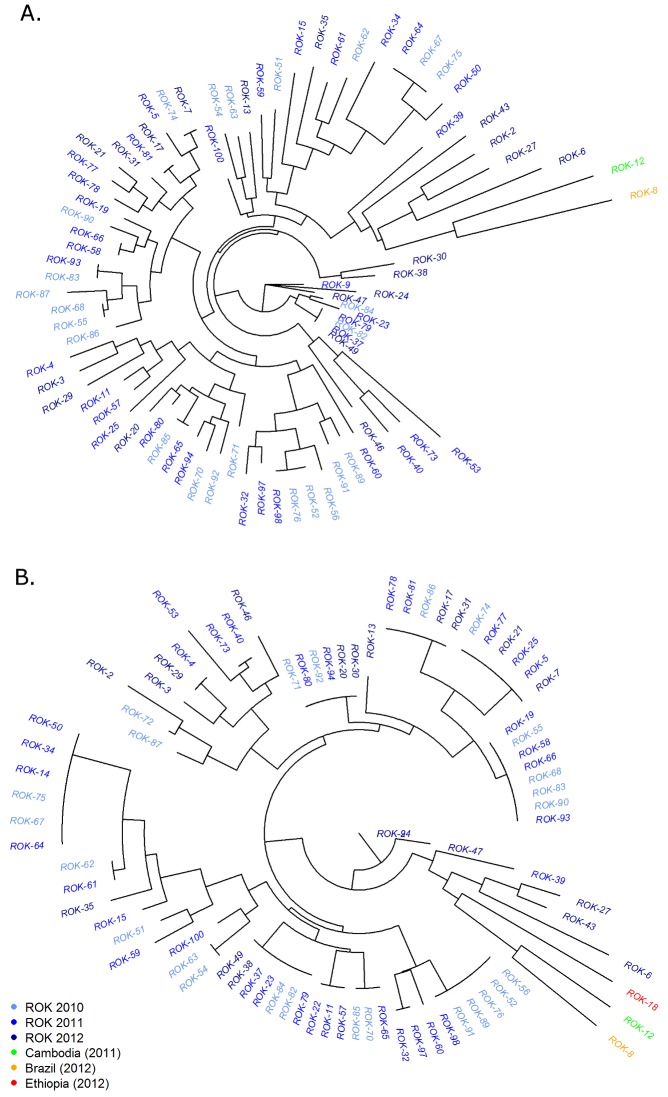
Unrooted neighbour-joining trees illustrating the genetic relatedness between *P*. *vivax* isolates in different years. Results derived from the 9 marker dataset in the top panel (A), and the 5 marker dataset in the bottom panel (B). N = 79 and 83 in panels A and B respectively.

Of the 77 ROK samples with complete data across the 9 loci, 64 distinct MLGs were observed, 55 (86%) of which were observed once, 6 (9%) twice, 2 (3%) three times, and 1 (1.5%) four times. Broken down by year, 21, 32 and 19 distinct MLGs were observed in 2010, 2011 and 2012 respectively. In each year, the majority of MLGs were only observed once; 18(86%) in 2010, 30 (94%) in 2011 and 19 (100%) in 2012. Seven of the nine (78%) multiply observed MLGs were present in more than one year, with two persisting from 2010 to 2012. The neighbour-joining tree in Fig 2 (panel A), illustrates that the 9-locus MLGs did not demonstrate any notable clustering by year of collection. This pattern was maintained when neighbour-joining analysis was restricted to the 5 balanced markers (Fig 2, panel B).

### Population Differentiation and Structure

Using both the 9 marker and 5 marker datasets, there was no evidence of genetic differentiation between any of the study years (*F*_ST_ range: -0.006–0.030; *F*’_ST_ range: -0.010–0.067) (S3 Table).

Using the *delta K* method to assess the output of STRUCTURE analysis in the 9 marker dataset, the most likely number of sub-populations was identified as 3 or 5, although the *delta K* for both estimates was low (*delta K* = 12 and 13 respectively) (S1 Fig). At *K* = 2, there was no difference in population structure between the three study years (S2 Fig). At *K* = 5, there was an increase in the number of isolates with high ancestry (≥70%; white colour code in S2 Fig) from 2010 (*n* = 0) to 2011 and 12 (*n* = 10).The isolates in the *K*5 cluster also exhibited moderate divergence relative to the majority of the ROK isolates, and several clustered with the three imported cases.

When STRUCTURE analysis was restricted to the 5 balanced markers, the *delta K* method identified *K* = 2 as the most likely number of sub-populations (*delta K* = 44) (S2 Fig). The 5 marker neighbour-joining tree provided in the supplementary material (S3 Fig), demonstrates that the *K*2 sub-population comprised of more diverged isolates relative to the *K*1 sub-population. However, there were no notable differences in the proportions of *K*1 and *K*2 between the study years (S4 Fig).

### Linkage disequilibrium

The overall level of LD was moderate but significant (*I*_A_^S^ = 0.098, *P*<0.05) (Table 4). When stratified by year, there was a trend of decreasing LD from 2010 (*I*_A_^S^ = 0.151, *P*<0.05) to 2011 (*I*_A_^S^ = 0.094, *P*<0.05) and 2012 (*I*_A_^S^ = 0.049, *P*<0.05). When the analysis was restricted to the 5 balanced markers, the decrease in LD was observed from 2010 (*I*_A_^S^ = 0.118, *P*<0.05) to 2011 (*I*_A_^S^ = 0.070, *P*<0.05) but not 2012 (*I*_A_^S^ = 0.078, *P*<0.05), possibly reflecting the combination of limited number of markers and sample size in the latter year (Table 4). After adjusting for multiply observed MLGs, LD remained significant in all years when the 9 marker set was applied. However, the temporal trend was no longer apparent using the 5 marker set, likely reflecting a limited ability to resolve distinct infections. Comparison of the current data with that from ROK studies of earlier years [8, 10] suggested an overall trend of declining LD from 1994 to 2013 (Table 3).

**Table 4:** Linkage disequilibrium.

		All infections ^1^		Unique MLGs ^2^	
Year	Marker Set	*N*	*I*_A_^S^	*N*	*I*_A_^S^
2010	^3^ 9 MS	24	0.151*	21 (88%)	0.106*
	^4^ 5 MS	25	0.118*	13 (52%)	0.0111*
2011	^3^ 9 MS	34	0.094*	32 (94%)	0.089*
	^4^ 5 MS	36	0.070*	20 (56%)	-0.035 ^NS^
2012	^3^ 9 MS	19	0.049*	19 (100%)	0.049*
	^4^ 5 MS	19	0.078*	15 (79%)	0.026*
2010–13	^3^ 9 MS	77	0.098*	64 (83%)	0.070*
	^4^ 5 MS	80	0.082*	33 (38%)	-0.013 ^NS^

^**1**^Only samples with no missing data at all 8 loci are included in the analyses. Note, all samples exhibited low complexity, hence there was no need to repeat the analysis with samples restricted to a maximum of one multi-allelic locus.

^2^ Unique set of multi-locus genotypes.

^3^ All 9 markers used in analysis.

^4^ Analysis restricted to 5 markers defined as balanced by Sutton [35]: MS1, MS5, MS10, MS12, MS20.

*0.01 < *P* < 0.05.

^NS^ Not significant (*P* > 0.05).

## Discussion

Using isolates sourced between 2010 and 2012, our study presents the most recent STR-based analysis of *P*. *vivax* diversity and transmission in the ROK to date. Our results complement those from previous studies and provide further evidence of increasing population diversity and declining LD over the years since re-emergence. Relative to other vivax-endemic regions, the diversity of *P*. *vivax* in the ROK between 2010 and 2013 has remained moderately low, LD is moderate but significant and polyclonal infections are rare. Sub-structure was apparent in the population, with strongest evidence of 2 co-circulating sub-populations. We discuss the possible epidemiological processes driving these patterns of diversity and structure.

Since re-emergence in 1993, vivax malaria in the ROK has largely been confined to regions adjacent to the DMZ, raising suggestion that the DPRK is a major reservoir of infection. *Anopheles sinensis* has been shown to travel up to 12 km in a single night, theoretically permitting traversal across the approximately 4 km wide DMZ [47]. Furthermore, ROK travellers are allowed to visit certain regions of the DPRK including Kaesong and Kumgang san. Incidence data also provides links between the vivax situation in the ROK and DPRK. In the early 2000s, the DPRK experienced its highest reported incidence of malaria, peaking at approximately 150,000 cases: this coincided with a peak incidence in the ROK, with over 4,000 cases reported [48]. Shortly after this period, the ROK began donating funds via the World Health Organization to the DPRK to aid malaria control efforts, and the incidence of vivax malaria declined in both the DPRK and the ROK [48]. Besides the potential influence of direct introductions from the DPRK, local transmission events have likely also had moderate impact on the genetic structure of the ROK vivax population in recent years as the proportion of civilian cases has increased.

The most recent peak incidence in the ROK was observed in 2010 (1,722 cases) followed by a decline to 838 in 2011 and 555 in 2012 [48]. Our analyses on samples collected during these years demonstrated an overall expected heterozygosity of 0.56. In the majority of vivax-endemic countries investigated to date, *H*_E_ frequently exceeds 0.8 [13, 14, 16, 19, 21–23, 25, 26, 29]. In this respect, the diversity observed in the ROK can be considered moderately low, with levels most comparable to low endemic settings in Sabah, Malaysia (*H*_E_ 0.58–0.67) [11] and Loreto, Peru (*H*_E_ 0.44–0.69) [30], inferring low transmission within the ROK.

At 3%, the prevalence of polyclonal infections between 2010 and 12 was also low. Indeed, even after reducing the threshold for calling minor alleles to 10% intensity of the major clone, only 6% of infections were polyclonal. Caution is advised in inter-study comparisons of polyclonality as the number and nature of markers as well as methods used for calling minor alleles is recognised to play a major role in the detection of polyclonal infections [49]. However, comparison is possible with APMEN studies using similar methodologies which reported polyclonality ranging from 4–12% in Central China [23], 26% in Malaysia [11], 30% in the Solomon Islands [14], 8–67% in Ethiopia [13], and 23–70% in Indonesia [25]. The low prevalence of polyclonal infections in the ROK demonstrated greatest comparability to Central China. These observations are consistent with a major contribution of the nature of relapse in shaping within-host infection diversity in *P*. *vivax*, the latency from ROK and Central China being less frequent with longer latency than the other studies so far reported. Low incidence and accordingly low rates of superinfection may also facilitate low rates of polyclonal infection.

Across 2010–12, significant LD was observed in the ROK, with a moderate index of association (*I*_A_^S^ = 0.1, *P*<0.05) relative to other geographic regions. *I*_A_^S^ <0.01 with no evidence of LD has been observed in parts of Indonesia [25], Papua New Guinea [22] and Ethiopia [13], and at the other extreme, significant LD with *I*_A_^S^ >0.25 has been observed in Sabah [11], Central China [23], and parts of Peru [30]. The mechanisms shaping LD in *P*. *vivax* remain poorly understood. In *P*. *falciparum* populations, genetic diversity tends to be reduced and LD enhanced in low transmission settings [50, 51]. This pattern is postulated to reflect the predominance of monoclonal infections in low transmission settings and consequently greater frequency of inbreeding. However, several *P*. *falciparum* studies and a large number of *P*. *vivax* studies have demonstrated significant LD in the presence of high population diversity and frequent polyclonal infections, leading to suggestions that other processes might also shape LD in *Plasmodium* (reviewed in [52]).

The paucity of polyclonal infections in the ROK supports largely clonal propagation dynamics, although the modest LD infers only a moderate degree of inbreeding. We considered that this unusual dynamics might reflect underestimation of the extent of LD in the ROK owing to features of the markers applied. Indeed, during mitotic replication, strand-slippage events may occur moderately frequently in microsatellites rendering them less stable than SNPs. However, the aforementioned studies in Sabah and Central China were able to detect strong LD using the same marker sets [11, 23]. Further, although the microsatellite panel was not identical to the panel applied here (5 shared markers), Iwagami and colleagues demonstrated the persistence of alleles for up to 10 years in the ROK, inferring moderate stability [9]. Additional studies using neutral SNP-based markers are nonetheless needed to confirm the genetic structure of *P*. *vivax* in the ROK.

We also considered that we may have underestimated the frequency of polyclonal infections in the ROK. On reducing the threshold for calling minor alleles from 33% to 10%, the prevalence of polyclonal infections remained low at 6%. Whilst we might identify more clones at lower relative thresholds, the chances of minor clones being sampled in a mosquito blood meal and consequent recombination would decline. Genotyping at additional markers would likely also reveal more polyclonal infections. By definition, however, the clones within the infection would be highly related. Further, as demonstrated in Indonesia, with 70% polyclonal infections in Sumba [25], the APMEN marker set has high potential to detect polyclonal infections.

Another explanation for the modest LD is that parasites introduced from an external reservoir(s) have enhanced the number of apparent recombination events in the ROK. Indeed, several previous genetic studies in the ROK have reached the conclusion that the extant vivax population is heavily shaped by continuous introductions from the DPRK [9, 10, 53]. It should be noted that this scenario does not exclude that local transmission events may also occur in the ROK and thus also contribute to the population structure. Importantly, this explanation allows for local propagation to take place largely by inbreeding, concordant with the low incidence, low prevalence of polyclonal infections, and limited period (July-August) of opportunity for recombination in the *A*. *sinensis* vector. This scenario also helps to explain the longitudinal trends in the genetic diversity and structure of *P*. *vivax* in the ROK described further below.

Despite the fluctuations in *P*. *vivax* incidence in the ROK, the data from this and other studies demonstrates a consistent rise in population diversity associated with a decline in LD over the years [8, 10]. Although the increase in expected heterozygosity between 2010 and 2012 in our study did not reach significance, the trend of increasing diversity in recent years is consistent with observations from independent studies using both neutral [8, 10] and surface protein markers [53–56]. The increasing diversity and declining LD might in part reflect an increase in local outbreeding rates. A recent study using sequence data generated at the *P*. *vivax* merozoite surface protein-1 gene (*PvMSP1*) demonstrated a gradual increase in the frequency of PvMSP1 recombinant types between 1996 and 2013, inferring increased outbreeding [57]. Indeed, the increased proportion of cases in the civilian population might have facilitated the dissemination of infections and potential for local outbreeding. However, as mentioned previously, the paucity of polyclonal infections restrains the extent of outbreeding in the ROK. Further, despite the large number of distinct MLGs (*n* = 64), the majority of MLGs that were observed more than once (7/9, 78%) persisted across two to three years without being broken down by recombination. An increase in the rates of local outbreeding alone is therefore unlikely to have led to the increased divergence in the parasite population.

An additional factor that may have facilitated the increase in population diversity and decline in LD is introductions of cases from an external reservoir(s). Indeed, the declining incidence of vivax cases in the ROK may have been associated with an increase in the relative proportion of externally sourced cases. As per earlier discussions, the introduction of cases from an external reservoir would permit diversity to increase in the ROK without the need for extensive outbreeding to take place locally. This scenario would therefore not conflict with the low rate of polyclonal infections observed or low incidence. Indeed, a recent study in the pre-elimination setting of Sri Lanka identified a similar paradox of increasing *P*. *vivax* diversity as local transmission levels declined: the authors concluded that this reflected increased diversity from imported infections [15]. The number of overseas imported cases reported in the ROK remains low (below 50 per year) despite a modest increase in the past few years [48]. However, these figures do not account for the burden of infections from the DPRK, which is less easily measured, and more likely to be a reservoir to the ROK. However, this remains speculative: genetic data on the DPRK vivax population is needed to gain better insight into the extent of genetic exchange with the ROK population.

Whilst STR markers are not ideal for investigating parasite geographic origin (mitochondrial data being preferable in most endemic regions [58]), we observed that, with the exception of a single isolate, all of the ROK infections carried a short MS20 allelic form (142 or 149 bp). Neither of the short MS20 alleles were observed in any of the imported cases investigated in this study or in our other studies conducted in Malaysia [11], Indonesia [25], Ethiopia [13] or Bhutan (manuscript under review) despite moderate to high diversity at the locus in all of these sites (*H*_*E*_ range: 0.69–0.92), demonstrating potential population-specificity of these alleles. It remains to be confirmed whether the short MS20 allelic form is found in the DPRK and whether it is exclusive to the Korean Peninsula. Nonetheless, the current evidence suggests that the MS20 locus is likely to be diverse in most other endemic regions and thus these regions are not likely to be major reservoirs of vivax malaria to the ROK. Rather, a restricted geographic region, most likely the DPRK given the epidemiological evidence, constitutes the main external reservoir of *P*. *vivax* infection to the ROK.

The ROK studies referenced in the temporal trends used different marker sets, with Honma *et al*. and Iwagami *et al*. applying 5/9 and 7/9, respectively, of the markers used in our current analysis. Nonetheless, the trend of increasing diversity and declining LD is replicated within the current study as well as that of Honma *et al*. and Iwagami *et al*., suggesting that the inter-study observations are unlikely to be an artefact of the different marker sets used.

Analysis of the local population structure using STRUCTURE software highlighted a greater capacity to detect subtle patterns of population structure when loci with excess diversity are excluded. Whilst *delta K* analysis of the 9 marker STRUCTURE output demonstrated weak evidence of 3 or 5 sub-populations in the ROK, when analysis was restricted to the 5 balanced markers, there was clear evidence for 2 sub-populations. Closer inspection of the isolates within each of the two sub-populations revealed that the isolates in the *K*2 cluster were generally more divergent than those in the *K*1 sub-population A previous study undertaken on *P*. *vivax* isolates collected in the ROK between 1994 and 2008 identified temporal changes in the local population structure reflecting increased diversity in the population in 2002 and 2003 [10]. We did not find any evidence of temporal trends in the distribution of the *K*1 and *K*2 isolates in our study, likely reflecting the limited time scale. Using mitochondrial sequence data, Iwagami and colleagues have previously postulated that two sub-populations observed in ROK isolates reflected long versus short latency infections on account of their clustering patterns in relation to other geographic isolates [59]. Owing to constraints in the availability of phenotypic data on infection latency, we were unable to assess this hypothesis in our study. Further studies with precisely defined latency and clinical phenotypes would lend additional insights, but it is likely that other epidemiological factors also underlie the observed structure.

## Conclusions

Whilst the underlying factors responsible for the trends observed in the diversity and structure of *P*. *vivax* in the ROK remain inconclusive, the available evidence suggests that introduced infections sourced from a single major reservoir of infection with moderate diversity has an important role in sustaining *P*. *vivax* infection in the ROK. These findings have critical implications for malaria control in this region and its elimination.

## Supporting Information

S1 Fig Delta K method: *ΔK* against *K*. Results derived from the 9 marker dataset in the top panel (A), and the 5 marker (balanced markers) dataset in the bottom panel (B). The 5 marker subset includes MS1, MS5, MS10, MS12 and MS20.
(TIF)

S2 Fig Population structure in 2010–12 using 9 marker dataset. Bar plot illustrating the population structure at the *K* clusters decided by *delta K* analysis: *K* = 2 (top) and *K* = 5 (bottom) using the 9 marker dataset. Each vertical bar represents an individual sample and each colour represents one of the *K* clusters (sub-populations) defined by STRUCTURE. For each sample, the predicted ancestry to each of the K sub-populations is represented by the colour-coded bars. *K*1 = light green, *K*2 = dark green, *K*3 = red, *K*4 = orange, and *K*5 = white.
(TIF)

S3 Fig Unrooted neighbour-joining tree using the 5 marker dataset with STRUCTURE-based colour-coding. Results derived from the 5 marker dataset (n = 83). Isolates with STRUCTURE-defined high ancestry (≥70%) to the *K*1 and *K*2 clusters at *K =* 2 in the 5 maker dataset are demarked in red and blue font respectively. Isolates with mixed ancestry to *K*1 and *K*2 and the three imported infections (not included in STRUCTURE analysis) are demarked in grey font.
(TIFF)

S4 Fig Population structure in 2010–12 using the 5 marker dataset. Bar plot illustrating the population structure at the *K* = 2 clusters decided by *delta K* analysis using the 5 marker dataset with grouping by study year. Each vertical bar represents an individual sample and each colour represents one of the *K* clusters (sub-populations) defined by STRUCTURE. For each sample, the predicted ancestry to each of the K sub-populations is represented by the colour-coded bars. *K*1 = light green, *K*2 = dark green.
(TIF)

S1 Table (CSV)

S2 Table Marker features in the ROK in 2010–13. (DOCX)

S3 Table Pair-wise differentiation between study years. ^1^ Analysis restricted to 5 markers defined as balanced by Sutton [35]: MS1, MS5, MS10, MS12, MS20. ***F***_**ST**_ (*P-value*) in lower left triangle. ***F***’_**ST**_ in upper right triangle.
(DOCX)

S4 Table MS20 diversity in APMEN studies. (DOCX)
