# Supplementary figures and images for: Further Evidence of Increasing Diversity of Plasmodium vivax in the Republic of Korea in Recent Years
Source: PLoS One. 2016 Mar 18;11(3):e0151514. doi: 10.1371/journal.pone.0151514 (PMC4798397; doi:10.1371/journal.pone.0151514)

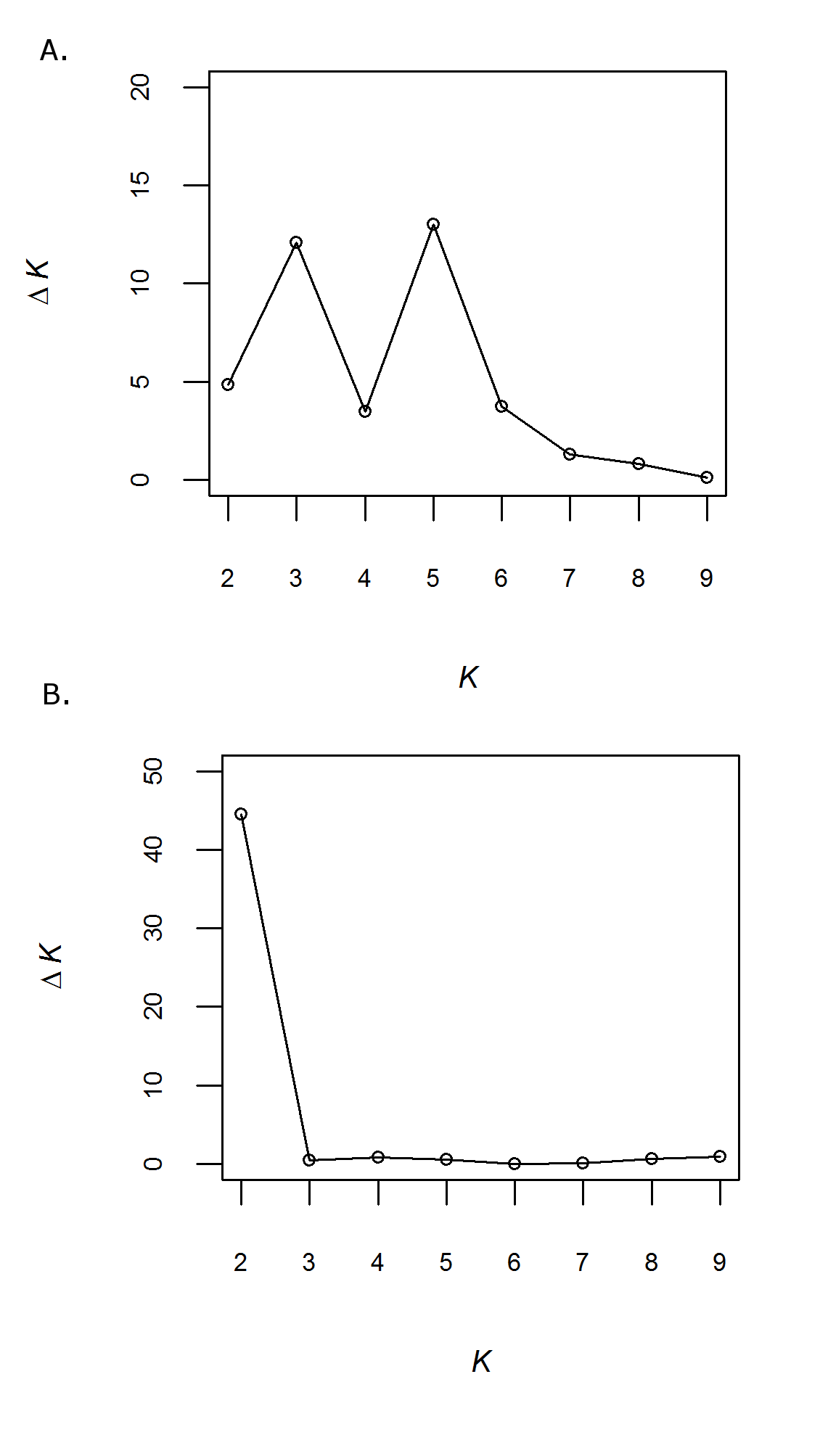

Supplement: S1 Fig — Results derived from the 9 marker dataset in the top panel (A), and the 5 marker (balanced markers) dataset in the bottom panel (B). The 5 marker subset includes MS1, MS5, MS10, MS12 and MS20. (TIF) [file pone.0151514.s001.tif]

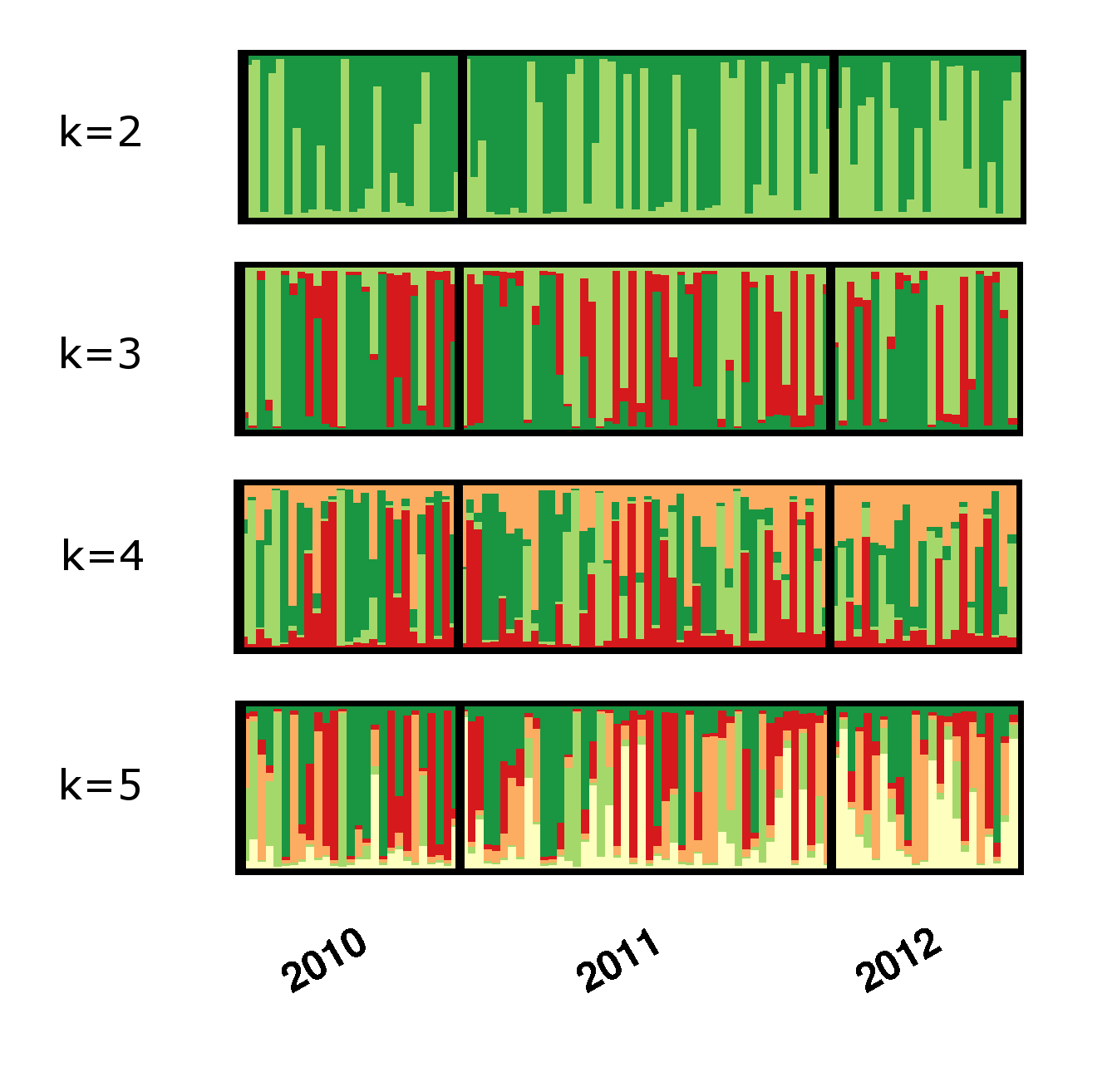

Supplement: S2 Fig — Bar plot illustrating the population structure at the K clusters decided by delta K analysis: K = 2 (top) and K = 5 (bottom) using the 9 marker dataset. Each vertical bar represents an individual sample and each colour represents one of the K clusters (sub-populations) defined by STRUCTURE. For each sample, the predicted ancestry to each of the K sub-populations is represented by the colour-coded bars. K1 = light green, K2 = dark green, K3 = red, K4 = orange, and K5 = white. (TIF) [file pone.0151514.s002.tif]

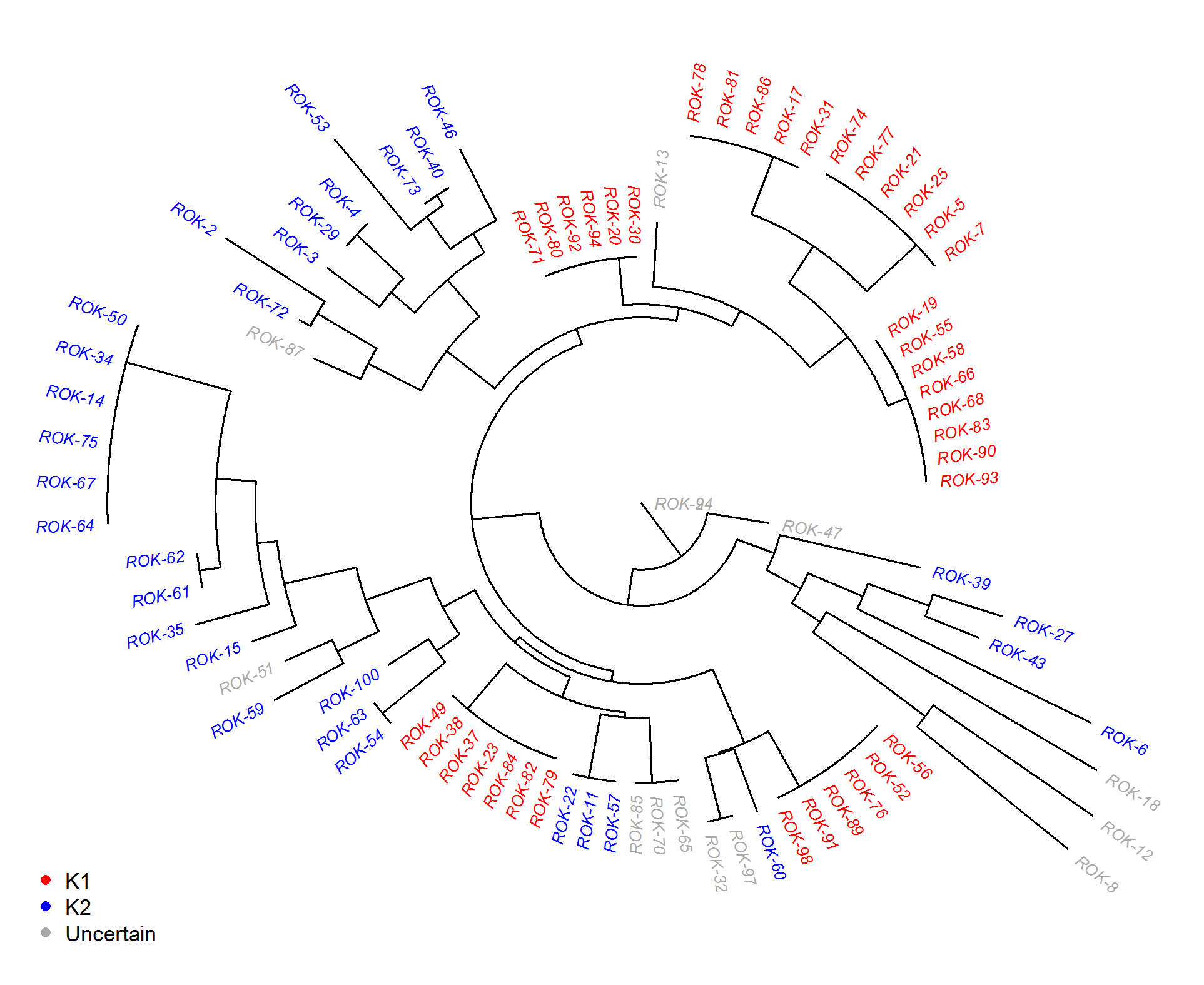

Supplement: S3 Fig — Results derived from the 5 marker dataset (n = 83). Isolates with STRUCTURE-defined high ancestry (≥70%) to the K1 and K2 clusters at K = 2 in the 5 maker dataset are demarked in red and blue font respectively. Isolates with mixed ancestry to K1 and K2 and the three imported infections (not included in STRUCTURE analysis) are demarked in grey font. (TIFF) [file pone.0151514.s003.tiff]

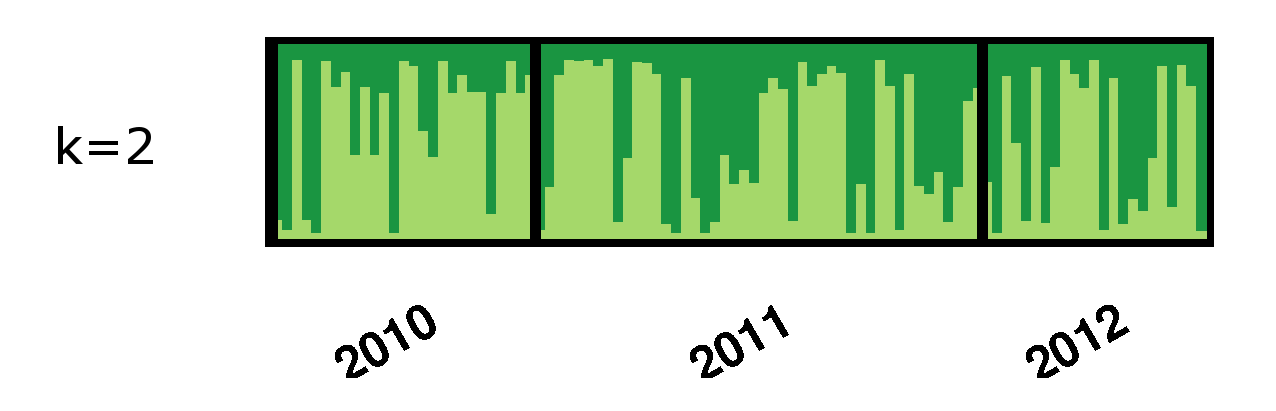

Supplement: S4 Fig — Bar plot illustrating the population structure at the K = 2 clusters decided by delta K analysis using the 5 marker dataset with grouping by study year. Each vertical bar represents an individual sample and each colour represents one of the K clusters (sub-populations) defined by STRUCTURE. For each sample, the predicted ancestry to each of the K sub-populations is represented by the colour-coded bars. K1 = light green, K2 = dark green. (TIF) [file pone.0151514.s004.tif]
