# Supplementary material for: Further Evidence of Increasing Diversity of Plasmodium vivax in the Republic of Korea in Recent Years
Source: PLoS One. 2016 Mar 18;11(3):e0151514. doi: 10.1371/journal.pone.0151514 (PMC4798397; doi:10.1371/journal.pone.0151514)
Supplement: S2 Table — (DOCX) [file pone.0151514.s006.docx]

**S2 Table. Marker features in the ROK in 2010-13**

| **Marker** | **Genotyping Fails, %** | **No. Alleles** | **Polyclonal Infections** |
| --- | --- | --- | --- |
| MS1 | 1 (1%) | 5 | 0 |
| MS10 | 6 (6%) | 3 | 0 |
| MS12 | 1 (1%) | 2 | 0 |
| MS16 | 0 (0%) | 13 | 1 |
| MS20 | 8 (8%) | 3 | 0 |
| MS5 | 1 (1%) | 7 | 0 |
| MS8 | 4 (4%) | 7 | 0 |
| msp1f3 | 1 (1%) | 5 | 0 |
| pv3.27 | 1 (1%) | 8 | 2 |
|  |  |  |  |
