# Supplementary material for: Further Evidence of Increasing Diversity of Plasmodium vivax in the Republic of Korea in Recent Years
Source: PLoS One. 2016 Mar 18;11(3):e0151514. doi: 10.1371/journal.pone.0151514 (PMC4798397; doi:10.1371/journal.pone.0151514)
Supplement: S3 Table — 1 Analysis restricted to 5 markers defined as balanced by Sutton [35]: MS1, MS5, MS10, MS12, MS20. FST (P-value) in lower left triangle. F’ST in upper right triangle. (DOCX) [file pone.0151514.s007.docx]

**S3 Table. Pair-wise differentiation between study years**

| **Year** | **Marker set** | **2010** | **2011** | **2012** |
| --- | --- | --- | --- | --- |
| **2010** | 9 MS | - | -0.001 | 0.067 |
|  | ^1^ 5 MS | - | -0.010 | 0.044 |
| **2011** | 9 MS | -0.000 (*P* = 0.405) | - | 0.013 |
|  | ^1^ 5 MS | -0.006 (*P* = 0.514) | - | 0.008 |
| **2012** | 9 MS | 0.030 (*P* = 0.099) | 0.005 (*P* = 0.252) | - |
|  | ^1^ 5 MS | 0.023 (*P* = 0.144) | 0.004 (*P* = 0.369) | - |

^1^ Analysis restricted to 5 markers defined as balanced by Sutton [[17](#_ENREF_17)]: MS1, MS5, MS10, MS12, MS20.

***F*_ST_** (*P-value*) in lower left triangle. ***F’*_ST_** in upper right triangle.
