# Supplementary material for: Further Evidence of Increasing Diversity of Plasmodium vivax in the Republic of Korea in Recent Years
Source: PLoS One. 2016 Mar 18;11(3):e0151514. doi: 10.1371/journal.pone.0151514 (PMC4798397; doi:10.1371/journal.pone.0151514)
Supplement: S4 Table — (DOCX) [file pone.0151514.s008.docx]

**S4 Table. MS20 diversity in APMEN studies**

| **Region** | **No. samples** | **Collection years** | **MS20 *H_E_*** | **MS20 allele range** | **Reference** |
| --- | --- | --- | --- | --- | --- |
| Republic of Korea* | 47 | 2011-12 | 0.04 | 142 - 175** | This study |
| Bhutan | 29 | 2013-14 | 0.90 | 175 - 230 | Under review |
| Sumba, Indonesia | 40 | 2012 | 0.85 | 185 - 215 | [[25](#_ENREF_25)] |
| Sabah, Malaysia | 90 | 2010-13 | 0.84 | 191 - 227 | [[11](#_ENREF_11)] |
| Kalimantan, Indonesia | 13 | 2012-13 | 0.90 | 191 - 227 | [[25](#_ENREF_25)] |
| Bangka, Indonesia | 86 | 2011 | 0.92 | 191 - 236 | [[25](#_ENREF_25)] |
| SNNPR, Ethiopia | 197 | 2013 | 0.90 | 194 - 239 | [[13](#_ENREF_13)] |
| West Timor, Indonesia | 29 | 2013 | 0.90 | 194 - 248 | [[25](#_ENREF_25)] |

* Not including 3 imported cases

** Only one sample with allele 175, all others 142-149

APMEN = Asia Pacific Malaria Elimination Network

SNNPR = South Nations, Nationalities and Peoples' Region

Note that APMEN studies in Central China [[23](#_ENREF_23)] and the Solomon Islands [[14](#_ENREF_14)] have not completed genotyping of MS20 to date
